# Behavioral unresponsiveness and impaired auditory event-related potentials in the anterior insula during rat absence seizures

**DOI:** 10.1101/2025.10.06.680740

**Published:** 2025-10-07

**Authors:** Stefan Sumsky, Rory Ashmeade, Jiayang Liu, Yang Zheng, Samiksha Chopra, Ben F. Gruenbaum, Cian McCafferty, Hal Blumenfeld

**Affiliations:** 1 Department of Neurology, Yale University School of Medicine, 333 Cedar Street, New Haven, Connecticut 06520, USA; 2 Department of Neuroscience, Yale University School of Medicine, 333 Cedar Street, New Haven, Connecticut 06520, USA; 3 Department of Neurosurgery, Yale University School of Medicine, 333 Cedar Street, New Haven, Connecticut 06520, USA

**Keywords:** Consciousness, absence epilepsy, seizures, sensorimotor processing, anterior insula, cortex, Genetic absence epilepsy rats of Strasbourg (GAERS), electroencephalography (EEG)

## Abstract

Absence epilepsy is marked by sudden lapses in consciousness accompanied by spike–wave discharges (SWDs), yet the neural mechanisms underlying impaired sensorimotor processing in these episodes remain unresolved. Prior work has shown that normal-appearing signals can persist in primary sensory cortical areas during seizures, suggesting that impaired behavioral responsiveness may arise from disruptions in higher-order association cortex. To investigate this hypothesis, we combined behavioral testing with simultaneous local field potential recordings in Genetic Absence Epilepsy Rats from Strasbourg (GAERS). Rats were trained in an auditory conditioned response task, allowing comparison of tone-evoked responses during interictal baseline and ictal SWDs. We found that behavioral performance collapsed during SWDs, with correct responses falling from ~88% at baseline to <1% during SWDs (p < 0.001). Nevertheless, auditory event-related potentials in primary auditory cortex (Au1) during SWDs were not significantly decreased. In contrast, we identified a novel oscillatory evoked potential in the anterior insular cortex (AIC) that was robust in controls, attenuated in GAERS at baseline, and markedly reduced during SWDs. Notably, reductions in AIC response magnitude also occurred during satiated, unmotivated states, but waveform structure was preserved, distinguishing motivational modulation from seizure-related disruption. These results demonstrate that absence seizures selectively impair signals in the anterior insula rather than in primary auditory cortex, identifying the AIC as a potentially critical hub for gating auditory conscious awareness. Beyond refining models of seizure-related unconsciousness, the discovery of an insular oscillatory potential highlights a candidate biomarker and intervention target for absence epilepsy, with broader implications for understanding impaired consciousness in anesthesia, sleep, and brain injury.

## Introduction

Absence epilepsy is characterized by brief episodes of staring and unresponsiveness associated with generalized 3–4 Hz spike–wave discharges (SWDs) on the electroencephalogram[1, 2]. These seizures most often arise in childhood and are accompanied by transient impairment of consciousness[1, 3–6]. Classically, SWDs are thought to reflect pathologically synchronous oscillations in cortico-thalamic loops that disrupt information flow through the thalamus, blocking primary sensory input. Consistent with this view, human EEG and fMRI studies find robust thalamic activation and concomitant decreases in widespread cortical and default-mode network (DMN) activity during absence seizures[7–10]. However, emerging evidence suggests that stimulus-driven activity in primary sensory cortices can persist during SWDs. Visual and somatosensory event-related potentials (ERPs) remain largely intact in patients and in the Genetic Absence Epilepsy Rat from Strasbourg (GAERS) model during seizures[3, 11–15]. In GAERS and WAG/Rij rats, whisker stimuli evoke normal cortical firing even during SWDs, suggesting that the thalamocortical relay of sensory information is not fully blocked[3]. These findings indicate that absence seizures may impair conscious perception not by abolishing sensory throughput, but by disrupting higher-order cortical processing downstream.

The anterior insular cortex (AIC) is a candidate site for such higher-order gating. The AIC, as part of the limbic/paralimbic salience network, integrates interoceptive, emotional, and sensory information and signals the occurrence of behaviorally relevant events[16–21]. Functional imaging and lesion studies link the AIC to conscious awareness. During anesthesia-induced loss of consciousness, for example, verbal commands evoke responses in primary auditory cortex and thalamus but fail to activate the AIC[22]. Conversely, intact insular connectivity predicts recovery of consciousness after coma[23]. These observations suggest that AIC activity is critical for bringing sensory inputs to awareness, potentially by coordinating interactions between externally oriented (e.g. auditory) networks and internally oriented (e.g. default-mode) networks[24].

The GAERS strain provides an excellent model for investigating these mechanisms[16, 25, 26]. GAERS rats carry genetic predispositions for absence seizures. By adulthood, they develop frequent, bilateral 7–9 Hz SWDs with sudden behavioral arrest, closely matching human childhood absence epilepsy both electrographically and pharmacologically[10, 27, 28]. In GAERS, SWDs arise focally in somatosensory cortex and then generalize through corticothalamic networks[29, 30], and fMRI studies show that during SWDs GAERS exhibit thalamic activation alongside decreased fronto-parietal and default-mode activity, similar to human absence seizures[4, 31]. Because GAERS seizures occur intermittently in otherwise alert animals, this model permits paired comparison of cortical sensory processing during interictal versus ictal periods using invasive recordings and behavioral assays.

Despite these previous studies and the advantages of absence models, the neural basis of impaired awareness in absence seizures remains unclear. In particular, it is not known where or how sensorimotor processing is disrupted during SWDs, leading to impaired behavioral responsiveness to external sensory stimuli. In this study, we addressed this gap by examining auditory stimulus-evoked activity in primary auditory cortex (Au1) and AIC of freely behaving GAERS versus non-epileptic control rats performing a simple auditory response task. We discovered a novel oscillatory evoked potential in the AIC: in control non-epileptic Wistar rats, auditory tone stimuli evoked a stable, multi-cycle response in AIC that matched the stimulus duration. Strikingly, this AIC oscillation was present but attenuated interictally in GAERS, and it collapsed nearly completely during SWDs. The AIC oscillation was also reduced when animals were satiated and no longer responded to auditory stimuli. In contrast, evoked responses in Au1 were preserved across conditions and animals. These findings suggest that absence seizures specifically disrupt an insula-dependent pathway for auditory awareness, unveiling a previously unrecognized electrophysiological signature (an oscillatory AIC potential) of conscious sensory processing.

## Methods

### Animals

All procedures were approved by the Yale University Institutional Animal Care and Use Committee and conducted in accordance with institutional and NIH guidelines. We used Genetic Absence Epilepsy Rats from Strasbourg (GAERS; 5 males, 5 females), a validated polygenic model of absence epilepsy, and Wistar rats (5 males, 5 females), the progenitor control strain. Animals were between 3 and 6 months of age at the time of study. Rats were housed on a 12 h light/dark cycle with *ad libitum* access to food and water unless otherwise specified. To prevent damage to implants and ensure recovery, rats were group housed prior to surgery and housed individually following electrode implantation.

### Surgical procedures

Animals underwent stereotaxic implantation of electroencephalography (EEG) screw electrodes, and bipolar local field potential (LFP) electrodes targeting primary auditory cortex (Au1) and anterior insular cortex (AIC). Anesthesia was induced with 5% isoflurane in oxygen, then maintained with ketamine/xylazine. Perioperative analgesia was provided with sustained-release buprenorphine and carprofen (5 mg/kg every 24 h, starting 1 h prior to surgery and for 48 h postoperatively). Following a 5-day recovery period, animals were retrained on the behavioral task to ensure performance stability before data collection commenced.

### Behavioral paradigm

**Figure 1.**
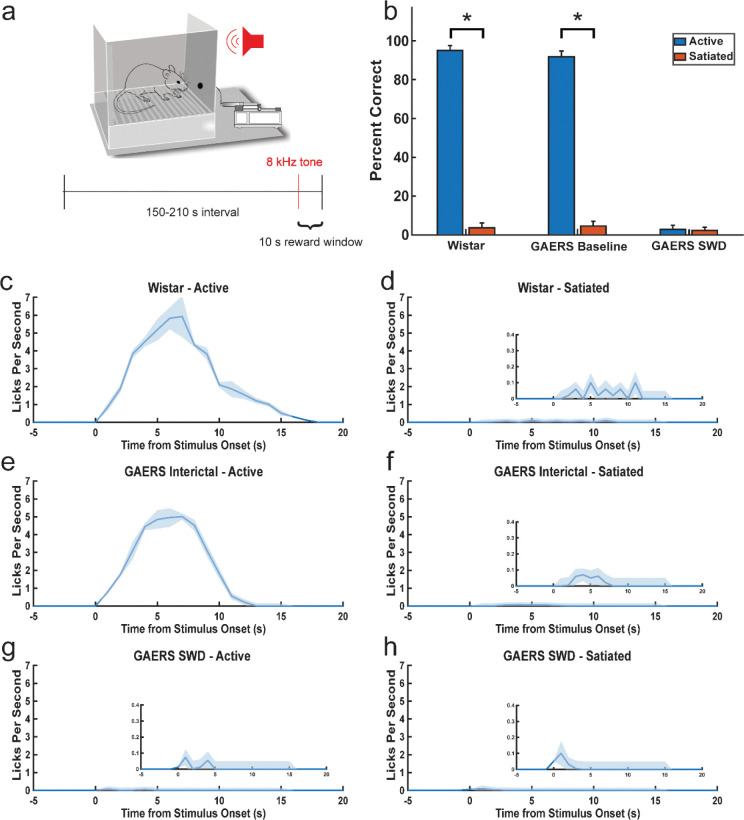
Absence seizures abolish auditory behavioral responses. (a) Schematic of the auditory tone response task in GAERS and Wistar rats. Rats licked a reward spout following 0.5 s, 8 kHz tones to receive sucrose reward. (b) Task performance across states. Correct response rates were high in GAERS interictal and Wistar controls but dropped from 88.2 ± 2.8% interictally to 0.4 ± 0.3% during SWDs (*p* < 0.001, Wilcoxon signed-rank, Bonferroni corrected; n = 10 GAERS). Responses recovered immediately post-SWD (78.2 ± 6.8%). (c–h) Lick-rate timecourses aligned to stimulus onset. Wistar controls (c,d) and GAERS interictal trials (e,f) showed rapid licking after tone onset, attenuated in satiated states. During SWDs (g,h), lick rates were nearly abolished.

To evaluate sensory processing during seizures, we adapted an auditory tone-detection response task from McCafferty et al. (2023). Rats were food restricted to 90% of baseline mass and trained to respond to a 0.5 s, 8 kHz auditory tone by licking a reward port within 10 s to receive a sucrose bolus (90 μL, 20% sucrose solution). Training was staged to gradually couple tone presentation to reward and shape conditioned responses. Performance criteria required animals to achieve ≥50 rewards in a 2 h session on two consecutive days before advancing to the next stage of. After training, tones were delivered at variable interstimulus intervals (150–210 s) or triggered by automated EEG detection of spike–wave discharges (SWDs)(Figure 1A). To minimize arousal-related seizure interruption, stimulus intensity was reduced to 45 dB, which pilot studies confirmed allowed SWDs to persist for >1 s after tone onset.

Behavioral events, auditory stimuli, and lick responses were recorded using Med-PC V software, with synchronization of TTL pulses to electrophysiological recordings. Correct responses were defined as licks occurring within 10 s of tone onset during the same behavioral state (interictal or ictal). Licks occurring after a state transition (e.g., ictal to interictal) were excluded from analysis.

### Electrophysiology

Bipolar LFP electrodes (.008/.2MM SS 2TW, Plastics One Inc). were implanted in Au1 (AP −4.8 mm, ML 7.0 mm, DV −4.5 mm) and AIC (AP +1.2 mm, ML 5.0 mm, DV −5.0 mm), with reference screws (0–80 × 3/32, PlasticsOne) placed epidurally over parietal cortex. Signals were amplified with Model 1800 Microelectrode AC Amplifier and Headstage (A-M Systems) between 0.1–1000 Hz bandpass and 1000× gain, and digitized with a Micro1401 acquisition unit (Cambridge Electronic Design) at 1 kHz. EEG and LFP data were synchronized with behavioral events via TTL pulse. SWDs were automatically detected in broadband EEG using an amplitude threshold (≥5–7 SD above baseline for ≥0.5 s), then visually confirmed for accuracy. Evoked potentials were time-locked to tone onset and averaged separately for interictal and ictal epochs.

### Statistical analysis

Behavioral performance (percent correct responses) and evoked potential magnitudes (V_RMS_) were compared across states (Wistar baseline, GAERS interictal, GAERS ictal; active vs satiated) using Wilcoxon signed-rank tests with Bonferroni correction for multiple comparisons. Lick-rate histograms were constructed in 0.5 s bins aligned to tone onset. Data are presented as mean ± SEM unless otherwise indicated. Statistical significance was set at p < 0.05.

## Results

### SWDs abolish auditory behavioral responses

**Figure 2.**
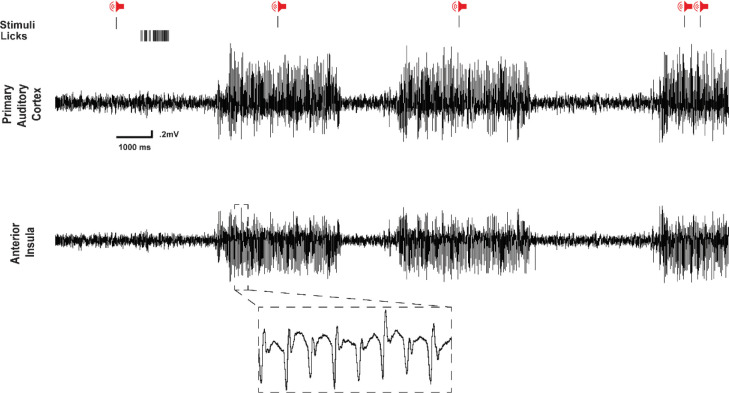
Representative electrophysiological traces during auditory task performance. Example GAERS recordings showing broadband EEG with identified SWDs (shaded), tone onset (vertical lines), lick responses, and LFP from Au1 and AIC. Stimuli during interictal periods evoked robust licking, while during SWDs no licking occurred. Typical spike–wave morphology is visible in EEG and LFP during ictal events.

Behavioral performance was robust during baseline in both GAERS and Wistar rats but collapsed during SWDs. Across 10 GAERS rats (n = 1482 stimuli), percent correct responses were 88.2 ± 2.8% interictally. During SWDs (n = 1156 stimuli), performance fell to 0.4 ± 0.3% (p < .001, Wilcoxon signed-rank, Bonferroni corrected; Fig. 2; Fig. 1b). Immediately after SWD termination, responses recovered to 78.2 ± 6.8%, not significantly different from pre-seizure baseline (p = 0.2163). These data indicate a reversible, seizure-specific impairment of auditory-driven behavior.

Lick-rate analyses reinforced these findings. In Wistar controls, tones evoked peaks of ~6 licks/s within 5 s of onset in the active state (Fig. 1c), whereas satiated states reduced responses to sporadic low-frequency licks (Fig. 1d). GAERS rats interictally mirrored this pattern (Fig. 1e–f). During SWDs, however, licking responses were nearly absent across both active and satiated conditions, with only minimal, auditory-induced activity detected (Fig.2; Fig. 1g–h).

### Event-related potentials in primary auditory cortex are preserved during SWDs

**Figure 3 |:**
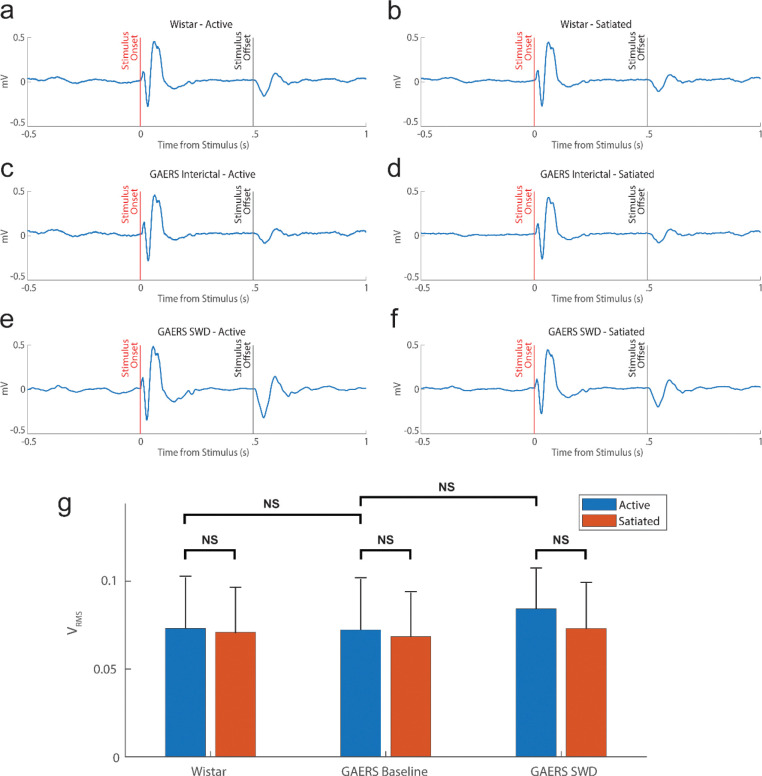
Event-related potentials in primary auditory cortex are preserved during SWDs. (a–f) Grand-averaged Au1 event-related potentials (ERPs) to tone onset across conditions. Sharp stereotyped onset responses were evident in Wistar and GAERS animals under both interictal and ictal states. (g) Quantification of Au1 response magnitudes (V_RMS_) revealed no significant differences across groups or states, confirming intact thalamocortical primary sensory transmission (*p* > 0.05, Wilcoxon signed-rank, Bonferroni corrected).

We next examined event-related potentials in Au1 to determine whether primary auditory processing was disrupted. In both Wistar and GAERS rats, interictal auditory event-related potentials displayed sharp, stereotyped onset responses. Intriguingly, these responses remained intact during SWDs (Fig. 3a–f). Quantitative comparison of V_RMS_ values revealed no significant differences across states (Fig. 3g), indicating preserved thalamocortical sensory input even during ictal events. Significance values for all pairs are reported in Supplementary Table S1. Thus, primary auditory responses in Au1 are not sufficient to explain the observed behavioral impairment.

### Novel AIC oscillatory responses are selectively disrupted during SWDs

**Figure 4.**
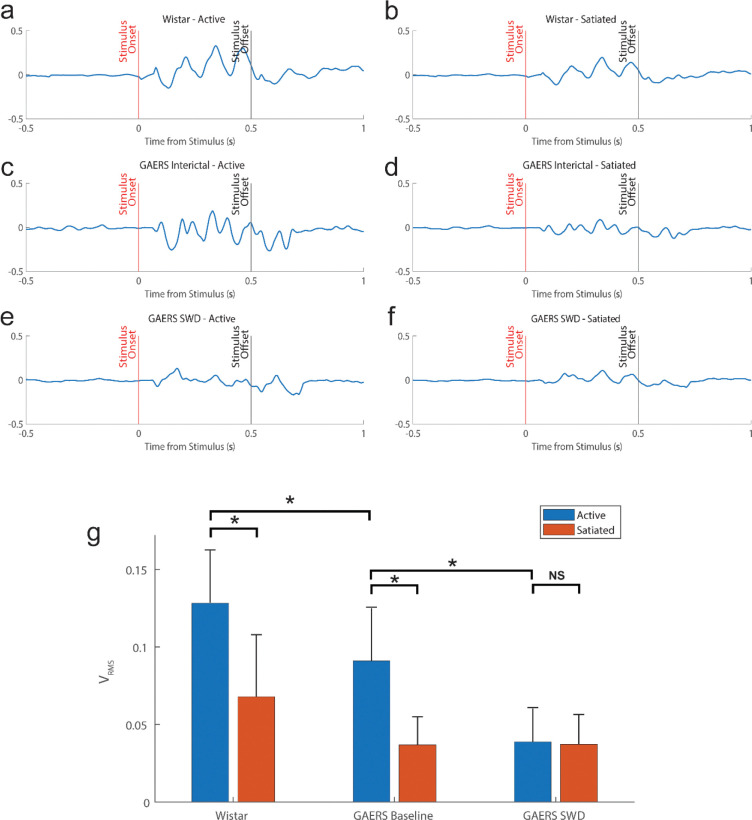
Novel oscillatory auditory responses in anterior insular cortex are selectively disrupted during SWDs. (a–d) Tone-evoked AIC responses in Wistar (a,b) and GAERS interictal states (c,d) displayed structured polyphasic oscillations, attenuated in GAERS relative to controls. (e,f) During SWDs, AIC oscillatory responses collapsed into disorganized activity with markedly reduced amplitude. (g) Quantification of AIC response magnitude (V_RMS_). GAERS interictal responses were reduced relative to Wistar (*p* < 0.001), with further reductions during SWDs (*p* < 0.001). Satiated states also attenuated responses in both strains (*p* < 0.01). Data are mean ± SEM; n = 10 rats.

By contrast, evoked potentials in AIC showed striking disruption during SWDs. In Wistar rats, tone-evoked responses were characterized by stable polyphasic oscillations (Fig. 4a–b). GAERS rats displayed similar oscillations interictally, but with reduced amplitude and stability (Fig. 4c–d). During SWDs, however, AIC responses lost the structured oscillatory pattern seen at baseline (Fig. 4e–f). Quantitative analyses confirmed significant reductions in V_RMS_ for GAERS interictal relative to Wistar controls (p < 0.01, Wilcoxon signed-rank, Bonferroni corrected; Fig. 4g) and further decreases during SWDs relative to interictal baseline (p < 0.001, Wilcoxon signed-rank, Bonferroni corrected; Fig. 4g). Additionally, satiated states attenuated responses in both strains (p < 0.01, Wilcoxon signed-rank, Bonferroni corrected; Fig. 4g). Significance values for all pairs are reported in Supplementary Table S2. Together, these results demonstrate that while Au1 responses persist, AIC oscillatory activity is significantly impacted during SWDs, correlating with behavioral deficits.

## Discussion

Our results identify the anterior insular cortex (AIC) as a potentially critical nexus for auditory consciousness and sensorimotor processing in absence epilepsy. We found that GAERS rats performed a tone-detection and response task with high accuracy outside of seizures but failed to respond during SWDs (Fig. 1b). Correspondingly, the auditory stimulus reliably evoked robust local field potentials in Au1 both interictally and during SWDs, with no significant change in amplitude (V_RMS_) or waveform shape (Fig. 3g). By contrast, auditory stimulus-evoked potentials in the anterior insular cortex were dramatically altered by SWDs. In control rats, each stimulus elicited a characteristic multi-cycle oscillatory potential in AIC that persisted for the duration of the stimulus (Fig. 4a–d). In GAERS, this oscillation was already diminished during interictal periods and was virtually abolished during SWDs (Fig. 4e–f), resulting in a marked reduction of AIC response magnitude (V_RMS_) (Fig. 4g). Thus, despite intact thalamocortical auditory transmission (preserved Au1 ERPs), absence seizures selectively disrupted the insular response, reflecting a breakdown of higher-order auditory processing.

These observations dovetail with prior studies in GAERS and human absence epilepsy. Previous work has shown that primary cortical sensory responses often persist during SWDs despite marked behavioral unresponsiveness. For example, Charpier and colleagues reported that in human patients and GAERS rats, sensory-evoked potentials in visual or somatosensory cortex remain largely unchanged during seizures[3]. Consistent with our findings, they concluded that “the brain can still process external stimuli during absence seizures” and that loss of consciousness is not due to a failure of primary thalamocortical relay[3]. We likewise observed robust stimulus-locked activity in primary cortex during SWDs (Fig. 3), indicating that the thalamic relay of auditory input remains operative in rodent absence seizures as well. However, we further show that evoked activity in AIC, a key association cortex area, is significantly affected. The emergence of a stereotyped, sustained oscillatory response in AIC appears to require a normal network state, and is lost during SWDs (Fig. 4e–g). In this way, our data indicate a specific neural correlate of higher sensorimotor processing in AIC that is distinct from primary sensory processing.

The AIC is well positioned to mediate such gating of conscious perception. Anatomically, the insula receives input from primary sensory areas and limbic regions and projects to frontal attentional and motor networks[6, 16–18, 32]. Functionally, it acts as a hub that “marks salient events for additional processing” and toggles between brain networks[20, 21, 24, 33]. The insula and other sensory association regions are further implicated in both disruption and recovery of consciousness across diverse conditions[22, 34]. Lesion and imaging studies in stroke and traumatic brain injury have long shown that damage to insula-linked networks impairs awareness. In comatose patients, functional MRI studies demonstrate that recovery of consciousness is accompanied by strengthening of anterior insula connectivity to frontoparietal cortices. Conversely, insular lesions or deactivation occur in patients who fail to regain awareness[23]. Likewise, during normal sleep and anesthesia the insula appears to gate sensory awareness: auditory stimuli can still activate primary cortex during non-REM sleep, but cortical responses beyond primary areas are suppressed during slow-wave oscillations[12, 35, 36]. In sum, AIC and adjacent association cortices form a “hub” that integrates sensory inputs into conscious experience, and disruption of this hub can explain parallels between absence seizures, stroke, deep sleep, and anesthesia. Our findings imply that common circuit-level mechanisms (e.g. loss of insular activation) may underlie unresponsiveness in all these states.

In our task, the tone-evoked oscillation in AIC may reflect a mechanism by which the insula integrates auditory information with interoceptive/motivational state, preparing behavioral responses. The abolition of this oscillation during SWDs suggests a “breakdown” in insular integration: even though Au1 still encodes the tone, the signal may fail to engage the salience network, leading to missed behavioral responses. This idea is supported by our finding that AIC responses remained intact when animals were awake but satiated: during reduced motivation the oscillatory waveform persisted but its magnitude decreased (Fig. 4b,d,g), whereas during SWDs the waveform itself disappeared. In other words, reduced internal drive downscaled insula responsiveness without altering its temporal pattern, whereas the seizure disrupted the entire insular response pattern. Such a dissociation implies that the oscillatory AIC potential is a signature of “engaged” processing, sensitive to both arousal/motivation and to pathological network synchrony.

Our study adds to recent work on the neural dynamics of absence seizures. McCafferty et al. used large-scale neuronal recordings in GAERS to show that the dominant effect of SWDs is widespread cortical and thalamic suppression of firing[31, 37]. They found that in GAERS firing rates in cortex and thalamus actually dip 2–3 seconds before seizure onset and remain low overall during the seizure, despite rhythmic “spiking” bursts during each spike-wave cycle. These results converge with ours: global cortical output is muted during SWDs, yet local oscillatory dynamics are preserved (the SWD spikes) even as total firing falls. Our finding that the AIC evoked potential vanishes during SWDs is consistent with an altered cortical state: the seizure takes over the cortex with a SWD rhythm, precluding the emergence of the higher-frequency oscillation in insular circuits normally triggered by a tone. Crucially, however, Au1 remains capable of responding within the SWD epoch, indicating that thalamocortical sensory transmission is not shut off. Thus, absence seizures appear to impose an “occlusion” on information flow through higher-order cortex without preventing inputs from reaching primary cortex.

These observations should be viewed in the context of standing theories of sensory disruption during SWDs. The classical view held that pathological thalamocortical synchrony during SWDs blocks sensory information flow[38]. However, convergent evidence, particularly our own, now argues against a pure relay block. Consistent with Chipaux et al. and others, we find that primary sensory evoked responses are preserved, indicating that thalamic input reaches cortex[3, 14, 15]. Instead, conscious perception fails because of “downstream” deficits. Our data add specificity to this idea by pinpointing the AIC as one such downstream bottleneck. This complements EEG-fMRI studies showing frontal-parietal and cortical network deactivation during absence seizure[31, 37]. Importantly, unlike models in which cortical firing would paradoxically increase during seizures, we and others observe that the dominant effect of SWDs is widespread reductions in cortical activity. The preserved Au1 potentials amid global cortical suppression suggests that SWDs do not abolish all activity, but selectively depress associative processing.

The insula-salience perspective also helps explain the behavioral pattern of absence seizures. Absences are typically accompanied by behavioral arrest and failure to respond to external cues, much like a short, reversible coma but with eyes open. Functional imaging in absence patients often shows deactivation of a broad frontoparietal network and the DMN during SWDs[24, 38]. These networks overlap substantially with the salience network and executive control networks to which the AIC is connected[21, 39–41]. Our results suggest that the impaired conscious awareness in absence seizure may stem from a functional disconnection: the cortex is swamped by abnormal oscillations, and AIC is unable to broadcast sensory information (or its salience ‘flag’) to frontal executive areas. Under normal conditions, the AIC-generated oscillatory evoked potential might play a role in signaling “auditory salience,” but SWDs silence it.

The preservation of waveform structure alongside magnitude reductions in the satiated state provides an intriguing insight. Even when reward expectation/desire was low and evoked amplitudes were reduced, the AIC oscillation kept its multi-peaked form (Fig. 4b,d). This indicates that the basic circuit mechanism generating the AIC evoked potential was intact under low motivation, albeit with lower gain. One interpretation is that motivational or neuromodulatory signals (for example, cholinergic or limbic inputs) scale AIC responsiveness without altering the intrinsic oscillatory pattern. By contrast, SWDs may disrupt the precise timing of excitatory and inhibitory inputs needed to sustain the oscillation, abolishing the pattern altogether. In any case, our results highlight that the insula’s auditory response is contingent on the animal’s internal state and the global brain rhythm.

There are limitations to our approach. Our conclusions derive from local field potential (LFP) recordings in cortical areas. LFPs integrate activity over millimeters of tissue and do not distinguish cell types or laminar sources. Thus, the precise neural generators of the evoked oscillations we measured remain unclear. Future studies at cellular resolution will be important. Calcium imaging or high-density laminar probes could elucidate which neuronal subpopulations in AIC (e.g. excitatory versus inhibitory) fail to respond during SWDs. Similarly, optogenetic activation of insular neurons could test their causal role in maintaining arousal during SWDs. Additionally, evidence of network involvement in the process of sensory disruption raises the need for simultaneous recording across the auditory pathway and cortex to clarify exactly where and when information is lost, and the role of network structure in that loss. Calcium imaging and grid electrocorticography provide promising avenues for these investigations in animal models. Finally, our study examined a single sensory modality. It will be important to determine if other senses (e.g. visual or somatosensory) show an analogous insular breakdown.

Our findings have practical implications. First, the pronounced disruption of insular evoked oscillations suggests they could serve as a biomarker of absence seizure severity or treatment effect. Continuous monitoring of AIC responses (via depth EEG or source-localized scalp recordings) might help identify impending cognitive lapses. Second, the AIC emerges as a potential therapeutic target. Modulating insular activity (pharmacologically or electrically) could help maintain sensory awareness during seizures. Interventions that enhance arousal networks have shown promise in other impaired-consciousness conditions [42]. Finally, the parallels with sleep and stroke imply that strategies developed for absence epilepsy (e.g. closed-loop neuromodulation triggered by insular signals) might generalize to other disorders of consciousness. Overall, our study pinpoints the anterior insula as a critical hub for auditory consciousness in absence seizures. By combining behavioral, electrophysiological, and network perspectives, it suggests a unified mechanism, the silencing of associative cortex, that links absences with broader disturbances of consciousness.

In summary, we have identified a novel electrophysiological marker of higher-order auditory processing in the insular cortex that is selectively impaired during absence seizures. This supports a model in which SWDs spare primary sensory representation but sever the pathway to sensorimotor processing and conscious awareness, in part by disabling a salience-network process in AIC. Going forward, it will be important to determine whether similar oscillatory potentials exist in other modalities or cortical areas, and whether their disruption correlates with sensory unconsciousness in human absences. More broadly, our work emphasizes the role of the insular cortex in conscious perception: not only must sensory signals reach cortex, they must be actively “tagged” by networks like the AIC for conscious awareness to occur.

## Supplementary Material

Supplement 1
