# Supplementary material for: Behavioral unresponsiveness and impaired auditory event-related potentials in the anterior insula during rat absence seizures
Source: bioRxiv. 2025 Oct 7:2025.10.06.680740. Preprint. [Version 1] doi: 10.1101/2025.10.06.680740 (PMC12632520; doi:10.1101/2025.10.06.680740)
Supplement: Supplement 1 [file NIHPP2025.10.06.680740v1-supplement-1.pdf]

bioRxiv preprint doi: https://doi.org/10.1101/2025.10.06.680740; this version posted October 7, 2025. The copyright holder for this preprint (which was not certified by peer review) is the author/funder, who has granted bioRxiv a license to display the preprint in perpetuity. It is made available under aCC-BY 4.0 International license.

Supplementary Information

Table S1: Significance Values | Primary Auditory Cortex VRMS

	Wistar - Active	Wistar - Satiated	GAERS Interictal - Active	GAERS Interictal - Satiated	GAERS SWD - Active	GAERS SWD - Satiated
Wistar - Active						
Wistar - Satiated	0.677584958					
GAERS Interictal - Active	0.472675594	0.733729996				
GAERS Interictal - Satiated	0.909721889	0.909721889	0.677584958			
GAERS SWD - Active	0.79133678	0.472675594	0.27303634	0.570750388		
GAERS SWD - Satiated	0.969849977	0.909721889	0.677584958	0.909721889	0.570750388	

Table S2: Significance Values | Anterior Insula VRMS

	Wistar - Active	Wistar - Satiated	GAERS Interictal - Active	GAERS Interictal - Satiated	GAERS SWD - Active	GAERS SWD - Satiated
Wistar - Active						
Wistar - Satiated	0.000136005					
GAERS Interictal - Active	0.001321971	0.104933594				
GAERS Interictal - Satiated	1.10464E-10	2.06302E-05	5.20735E-06			
GAERS SWD - Active	2.65665E-09	0.000948645	1.97604E-05	0.566956861		
GAERS SWD - Satiated	4.64736E-10	0.000250877	1.45801E-05	0.444278031	0.980807732	
